# *catena*-Poly[(μ_2_-3-carb­oxy-5-nitro­benzoato)(μ_3_-5-nitro­benzene-1,3-dicarboxyl­ato)(1,10-phenanthroline)gadolinium(III)]

**DOI:** 10.1107/S1600536810035452

**Published:** 2010-09-08

**Authors:** Jun Wang, Zhi-Li Fang

**Affiliations:** a Zhongshan Polytechnic, Zhongshan, Guangdong 528404, People’s Republic of China; b School of Basic Science, East China Jiaotong University, Nanchang 330013, People’s Republic of China

## Abstract

The crystal structure of the title complex, [Gd(C_8_H_3_NO_6_)(C_8_H_4_NO_6_)(C_12_H_8_N_2_)]_*n*_, contains polymeric chains made up of Gd^III^ atoms, 1,10-phenanthroline and fully or half-deproton­ated 5-nitro­benzene-1,3-dicarb­oxy­lic acid (H_2_ *L*) ligands. The Gd^III^ atom is coordinated in a distorted bicapped trigonal-prismatic fashion by six O atoms from two H*L* ^−^ and three *L* ^2−^ ligands, and by two N atoms from the 1,10-phenanthroline ligand. The *L* ^2−^ ligands bridge the Gd–phenanthroline units, forming chains running parallel to [100]. O—H⋯O hydrogen bonding as well as π–π stacking inter­actions with an inter­planar distance of 3.599 (2) Å assemble neighboring polymeric chains.

## Related literature

For background to π–π stacking in biological systems, see: Deisenhofer & Michel (1989 ▶). For some crystal structures of metal complexes exhibiting π–π stacking, see: Li *et al.* (2005 ▶); Pan & Xu (2004 ▶); Wu *et al.* (2003 ▶); Qiu *et al.* (2009 ▶).
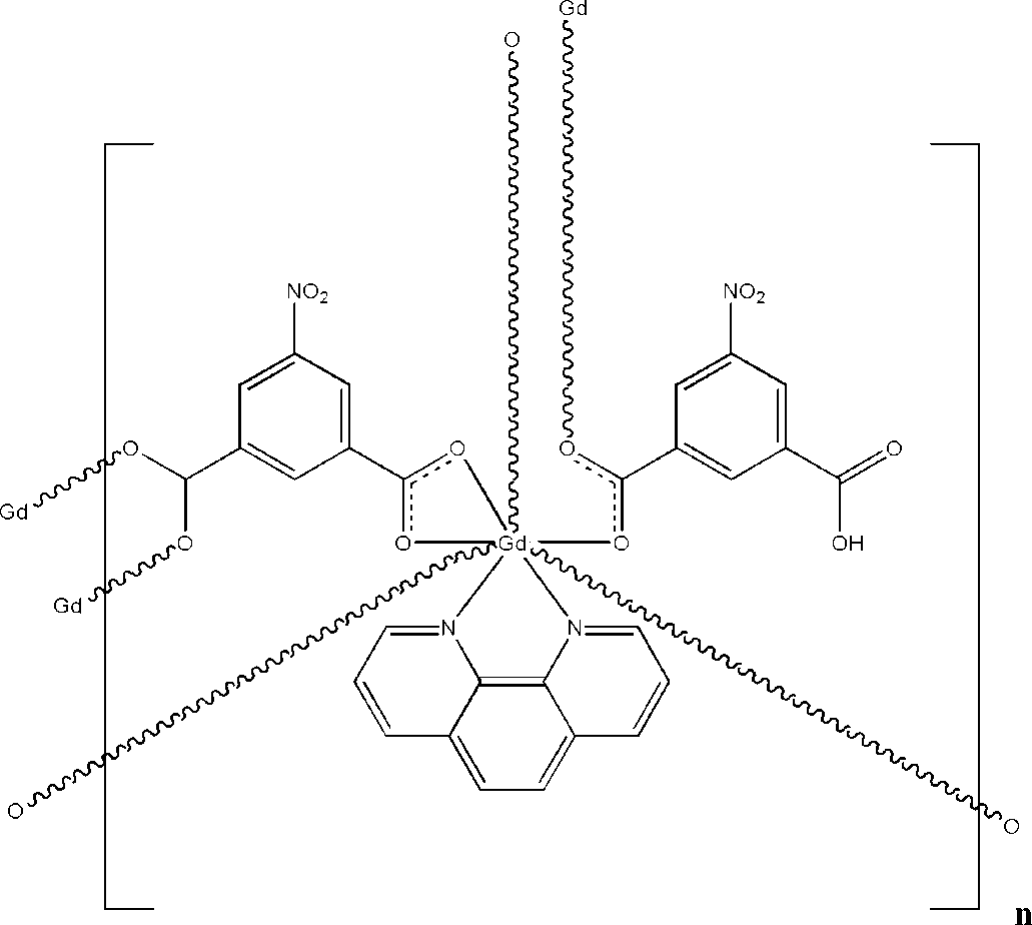


## Experimental

### 

#### Crystal data

[Gd(C_8_H_3_NO_6_)(C_8_H_4_NO_6_)(C_12_H_8_N_2_)]
*M* *_r_* = 756.69
Triclinic, 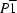

*a* = 10.300 (3) Å
*b* = 12.030 (3) Å
*c* = 12.150 (3) Å
α = 70.581 (4)°
β = 85.925 (4)°
γ = 76.512 (2)°
*V* = 1380.6 (7) Å^3^
*Z* = 2
Mo *K*α radiation
μ = 2.48 mm^−1^
*T* = 298 K
0.28 × 0.26 × 0.22 mm

#### Data collection

Bruker SMART CCD area-detector diffractometer
Absorption correction: multi-scan (*SADABS*; Sheldrick, 1996 ▶) *T*_min_ = 0.544, *T*_max_ = 0.612
6876 measured reflections
4837 independent reflections
4259 reflections with *I* > 2σ(*I*)
*R*_int_ = 0.030

#### Refinement

*R*[*F* ^2^ > 2σ(*F* ^2^)] = 0.045
*wR*(*F* ^2^) = 0.121
*S* = 1.04
4837 reflections
407 parameters
H-atom parameters constrained
Δρ_max_ = 2.51 e Å^−3^
Δρ_min_ = −2.59 e Å^−3^

### 

Data collection: *SMART* (Bruker, 2004 ▶); cell refinement: *SAINT-Plus* (Bruker, 2004 ▶); data reduction: *SAINT-Plus*; program(s) used to solve structure: *SHELXS97* (Sheldrick, 2008 ▶); program(s) used to refine structure: *SHELXL97* (Sheldrick, 2008 ▶); molecular graphics: *SHELXTL* (Sheldrick, 2008 ▶); software used to prepare material for publication: *SHELXTL*.

## Supplementary Material

Crystal structure: contains datablocks I, global. DOI: 10.1107/S1600536810035452/wm2394sup1.cif

Structure factors: contains datablocks I. DOI: 10.1107/S1600536810035452/wm2394Isup2.hkl

Additional supplementary materials:  crystallographic information; 3D view; checkCIF report

## Figures and Tables

**Table 1:** Hydrogen-bond geometry (Å, °)

*D*—H⋯*A*	*D*—H	H⋯*A*	*D*⋯*A*	*D*—H⋯*A*
O5—H5*A*⋯O7^i^	0.82	2.03	2.737 (7)	145

Symmetry code: (i) 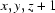.

**Fig. 1.**
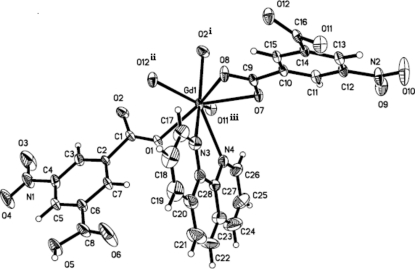
Part of the structure of (I), showing the coordination of the Gd atom with the atomic numbering scheme. Non-H atoms are shown with 30% probability displacement ellipsoids, H atoms as spheres of arbitrary radius. [Symmetry codes: (i) 2 - x, -y, 1 - z; (ii) 1 - x, y, 1 - z; (iii) 1 + x, y, z.]

**Fig. 2.**
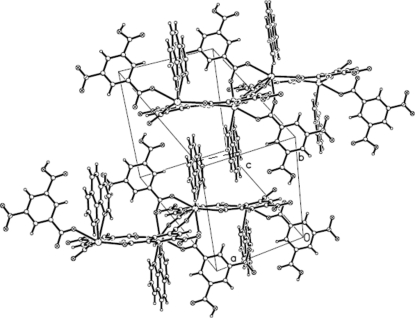
View of π–π interactions consolidating the three-dimensional network of (I).

## supplementary crystallographic information

### Comment

Recently, we became interested in the nature of π–π stacking as it plays an important role in some biological processes (Deisenhofer & Michel, 1989). A series of metal complexes incorporating different aromatic ligands has been prepared and their crystal structures provide useful information about π–π stacking (Wu *et al.*, 2003; Pan & Xu, 2004; Li *et al.*, 2005 Qiu *et al.*, 2009). As part of our ongoing investigations, the title complex, (I), incorporating 1,10-phenanthroline, has been prepared.

As depicted in Fig. 1, the Gd^III^ atom has a distorted bicapped trigonal-prismatic coordination, defined by six O atoms from two 1-carboxy-5-nitro-3-benzoate (H*L*^-^, where *L* is 5-nitro-1,3-benzenedicarboxyic acid) and three 5-nitro-1,3-benzenedicarboxylate ligands (*L*^2-^), and two N atoms from the 1,10-phenanthroline ligand. The *L*^2-^ ligands link Gd-phenanthroline moieties, forming an infinite polymeric chain running along [100]. The H*L*^-^ and *L*^2-^ ligands in this complex have two different coordination modes: the first ligand uses one of its carboxylate groups to link one Gd^III^ ions in a chelating coordination mode, the other carboxylate group links two Gd^III^ ions in a bis-monodentate coordination mode, whereas the other ligand uses its carboxylate groups to link two Gd^III^ ions in a bridging coordination mode. In the chain, the closest Gd···Gd separation is 4.333 (3) Å. These chains interact with each other by π–π stacking of adjacent phenanthroline groups (interplanar distance of 3.599 (2) Å). O—H···O hydrogen bonding interactions between carboxyl and carboxylate groups of neighbouring ligands help to consolidate the structure (Table 1; Fig. 2).

### Experimental

A sample of Gd_2_O_3_ (0.0732 g, 0.20 mmol), 5-nitro-1,3-benzenedicarboxylic acid (0.1015 g, 0.50 mmol), 1,10-phenanthroline (0.0991 g, 0.50 mmol) and distilled water (8 ml) were mixed in a Teflon-lined stainless steel vessel with 15 ml capacity. The mixture was heated under autogenous pressure at 393 K for 48 h and cooled slowly to room temperature.

### Refinement

The C— and O—bound H atoms were included in the riding-model approximation, with C—H = 0.97 Å and O—H = 0.82 Å, and *U*_iso_(H) = 1.2*U*_eq_(C) and 1.5*U*_eq_ (O). The highest and the deepest hole the final difference Fourier map are located 1.03 and 1.05 Å, respectively, from the Gd1 atom.

### Crystal data

**Table:**

[Gd(C_8_H_3_NO_6_)(C_8_H_4_NO_6_)(C_12_H_8_N_2_)]	*Z* = 2
*M**_r_* = 756.69	*F*(000) = 742
Triclinic, *P*1	*D*_x_ = 1.820 Mg m^−^^3^
Hall symbol: -P 1	Mo *K*α radiation, λ = 0.71073 Å
*a* = 10.300 (3) Å	Cell parameters from 5111 reflections
*b* = 12.030 (3) Å	θ = 2.6–27.7°
*c* = 12.150 (3) Å	µ = 2.48 mm^−^^1^
α = 70.581 (4)°	*T* = 298 K
β = 85.925 (4)°	Prism, colorless
γ = 76.512 (2)°	0.28 × 0.26 × 0.22 mm
*V* = 1380.6 (7) Å^3^	

### Data collection

**Table:**

Bruker SMART CCD area-detector diffractometer	4860 independent reflections
Radiation source: fine-focus sealed tube	4259 reflections with *I* > 2σ(*I*)
graphite	*R*_int_ = 0.030
φ and ω scan	θ_max_ = 25.3°, θ_min_ = 1.8°
Absorption correction: multi-scan (*SADABS*; Sheldrick, 1996)	*h* = −12→12
*T*_min_ = 0.544, *T*_max_ = 0.612	*k* = −13→14
6896 measured reflections	*l* = −14→13

### Refinement

**Table:**

Refinement on *F*^2^	Primary atom site location: structure-invariant direct methods
Least-squares matrix: full	Secondary atom site location: difference Fourier map
*R*[*F*^2^ > 2σ(*F*^2^)] = 0.045	Hydrogen site location: inferred from neighbouring sites
*wR*(*F*^2^) = 0.121	H-atom parameters constrained
*S* = 1.03	*w* = 1/[σ^2^(*F*_o_^2^) + (0.0811*P*)^2^] where *P* = (*F*_o_^2^ + 2*F*_c_^2^)/3
4837 reflections	(Δ/σ)_max_ < 0.001
407 parameters	Δρ_max_ = 2.51 e Å^−^^3^
0 restraints	Δρ_min_ = −2.59 e Å^−^^3^

### Special details

**Table:**

Geometry. All e.s.d.'s (except the e.s.d. in the dihedral angle between two l.s. planes) are estimated using the full covariance matrix. The cell e.s.d.'s are taken into account individually in the estimation of e.s.d.'s in distances, angles and torsion angles; correlations between e.s.d.'s in cell parameters are only used when they are defined by crystal symmetry. An approximate (isotropic) treatment of cell e.s.d.'s is used for estimating e.s.d.'s involving l.s. planes.
Refinement. Refinement of *F*^2^ against ALL reflections. The weighted *R*-factor *wR* and goodness of fit *S* are based on *F*^2^, conventional *R*-factors *R* are based on *F*, with *F* set to zero for negative *F*^2^. The threshold expression of *F*^2^ > σ(*F*^2^) is used only for calculating *R*-factors(gt) *etc*. and is not relevant to the choice of reflections for refinement. *R*-factors based on *F*^2^ are statistically about twice as large as those based on *F*, and *R*- factors based on ALL data will be even larger.

### Fractional atomic coordinates and isotropic or equivalent isotropic displacement parameters (Å^2^)

**Table:**

	*x*	*y*	*z*	*U*_iso_*/*U*_eq_	
C1	1.0366 (7)	0.0929 (6)	0.6625 (5)	0.0330 (14)	
C2	1.0511 (6)	0.1117 (6)	0.7766 (5)	0.0308 (13)	
C3	1.1673 (7)	0.0513 (6)	0.8416 (6)	0.0407 (16)	
H3	1.2371	0.0040	0.8132	0.049*	
C4	1.1762 (8)	0.0635 (7)	0.9504 (6)	0.0465 (18)	
C5	1.0753 (7)	0.1320 (6)	0.9960 (6)	0.0423 (16)	
H5	1.0841	0.1378	1.0694	0.051*	
C6	0.9615 (7)	0.1914 (7)	0.9309 (6)	0.0419 (16)	
C7	0.9488 (7)	0.1835 (6)	0.8200 (6)	0.0374 (15)	
H7	0.8723	0.2261	0.7755	0.045*	
C8	0.8451 (9)	0.2616 (8)	0.9788 (7)	0.057 (2)	
C9	0.5970 (6)	0.1875 (6)	0.3097 (5)	0.0292 (13)	
C10	0.4627 (6)	0.2185 (5)	0.2498 (5)	0.0270 (12)	
C11	0.4353 (6)	0.3072 (6)	0.1421 (5)	0.0321 (13)	
H11	0.5013	0.3440	0.0995	0.039*	
C12	0.3063 (6)	0.3388 (6)	0.1007 (5)	0.0328 (14)	
C13	0.2038 (6)	0.2870 (6)	0.1622 (5)	0.0314 (13)	
H13	0.1169	0.3128	0.1331	0.038*	
C14	0.2355 (6)	0.1959 (5)	0.2679 (5)	0.0256 (12)	
C15	0.3643 (6)	0.1606 (5)	0.3105 (5)	0.0279 (12)	
H15	0.3858	0.0976	0.3804	0.033*	
C17	0.5715 (9)	0.1824 (8)	0.6347 (8)	0.061 (2)	
H17	0.5663	0.1088	0.6280	0.073*	
C18	0.4954 (10)	0.2208 (11)	0.7209 (9)	0.085 (4)	
H18	0.4424	0.1727	0.7704	0.103*	
C19	0.4993 (10)	0.3254 (11)	0.7313 (9)	0.081 (3)	
H19	0.4484	0.3519	0.7878	0.097*	
C20	0.5823 (9)	0.3984 (9)	0.6552 (8)	0.064 (3)	
C21	0.5923 (12)	0.5114 (12)	0.6605 (11)	0.091 (4)	
H21	0.5419	0.5420	0.7148	0.109*	
C22	0.6719 (14)	0.5746 (11)	0.5897 (12)	0.096 (4)	
H22	0.6781	0.6476	0.5973	0.115*	
C23	0.7507 (11)	0.5337 (9)	0.4998 (10)	0.074 (3)	
C24	0.8317 (13)	0.5984 (10)	0.4198 (12)	0.095 (4)	
H24	0.8409	0.6722	0.4234	0.114*	
C25	0.8966 (12)	0.5558 (9)	0.3378 (11)	0.088 (3)	
H25	0.9508	0.5991	0.2843	0.106*	
C26	0.8809 (9)	0.4432 (7)	0.3339 (8)	0.057 (2)	
H26	0.9240	0.4151	0.2753	0.068*	
C27	0.7400 (8)	0.4227 (7)	0.4915 (7)	0.0451 (18)	
C28	0.6557 (7)	0.3526 (7)	0.5716 (6)	0.0447 (18)	
Gd1	0.82752 (3)	0.14792 (3)	0.44144 (2)	0.02604 (13)	
N1	1.2997 (8)	−0.0016 (8)	1.0209 (7)	0.072 (2)	
N2	0.2724 (6)	0.4320 (6)	−0.0147 (5)	0.0500 (16)	
N3	0.6506 (6)	0.2452 (5)	0.5622 (5)	0.0448 (15)	
N4	0.8089 (6)	0.3768 (5)	0.4088 (5)	0.0414 (14)	
O1	0.9505 (5)	0.1690 (4)	0.5913 (4)	0.0412 (11)	
O2	1.1124 (5)	0.0033 (5)	0.6459 (4)	0.0466 (12)	
O3	1.3864 (8)	−0.0642 (9)	0.9807 (8)	0.118 (4)	
O4	1.3055 (9)	0.0089 (8)	1.1154 (7)	0.113 (3)	
O5	0.8641 (6)	0.2486 (6)	1.0881 (5)	0.0647 (16)	
H5A	0.7931	0.2735	1.1164	0.097*	
O6	0.7481 (8)	0.3226 (11)	0.9241 (7)	0.140 (5)	
O7	0.6825 (4)	0.2498 (4)	0.2637 (4)	0.0335 (10)	
O8	0.6158 (4)	0.1067 (4)	0.4063 (4)	0.0413 (11)	
O9	0.3605 (6)	0.4789 (5)	−0.0694 (5)	0.0593 (15)	
O10	0.1591 (7)	0.4607 (8)	−0.0498 (6)	0.103 (3)	
C16	0.1297 (6)	0.1363 (6)	0.3374 (5)	0.0300 (13)	
O11	0.0114 (4)	0.1968 (4)	0.3252 (4)	0.0421 (11)	
O12	0.1665 (5)	0.0297 (4)	0.4038 (4)	0.0448 (12)	

### Atomic displacement parameters (Å^2^)

**Table:**

	*U*^11^	*U*^22^	*U*^33^	*U*^12^	*U*^13^	*U*^23^
C1	0.038 (4)	0.044 (4)	0.022 (3)	−0.013 (3)	−0.002 (3)	−0.014 (3)
C2	0.039 (4)	0.037 (3)	0.020 (3)	−0.010 (3)	−0.004 (3)	−0.012 (3)
C3	0.039 (4)	0.048 (4)	0.032 (4)	−0.001 (3)	−0.007 (3)	−0.013 (3)
C4	0.047 (4)	0.059 (5)	0.030 (4)	−0.007 (3)	−0.014 (3)	−0.010 (3)
C5	0.055 (4)	0.052 (4)	0.023 (3)	−0.011 (3)	−0.004 (3)	−0.016 (3)
C6	0.049 (4)	0.053 (4)	0.025 (4)	−0.010 (3)	0.001 (3)	−0.016 (3)
C7	0.043 (4)	0.052 (4)	0.020 (3)	−0.010 (3)	−0.002 (3)	−0.014 (3)
C8	0.055 (5)	0.080 (6)	0.041 (5)	0.001 (4)	0.000 (4)	−0.038 (4)
C9	0.019 (3)	0.044 (4)	0.024 (3)	−0.006 (2)	−0.001 (2)	−0.012 (3)
C10	0.020 (3)	0.039 (3)	0.021 (3)	−0.005 (2)	0.000 (2)	−0.009 (3)
C11	0.025 (3)	0.045 (4)	0.024 (3)	−0.011 (3)	0.004 (2)	−0.006 (3)
C12	0.038 (4)	0.041 (3)	0.017 (3)	−0.015 (3)	0.000 (3)	−0.002 (3)
C13	0.024 (3)	0.046 (4)	0.024 (3)	−0.007 (3)	−0.001 (2)	−0.011 (3)
C14	0.023 (3)	0.035 (3)	0.019 (3)	−0.008 (2)	0.001 (2)	−0.008 (2)
C15	0.027 (3)	0.036 (3)	0.019 (3)	−0.006 (2)	−0.002 (2)	−0.006 (3)
C17	0.054 (5)	0.060 (5)	0.057 (6)	−0.004 (4)	0.025 (4)	−0.014 (4)
C18	0.073 (7)	0.093 (8)	0.062 (7)	0.011 (6)	0.037 (5)	−0.014 (6)
C19	0.070 (7)	0.101 (8)	0.052 (6)	0.010 (6)	0.029 (5)	−0.024 (6)
C20	0.065 (6)	0.072 (6)	0.053 (5)	0.019 (4)	−0.007 (4)	−0.039 (5)
C21	0.084 (8)	0.117 (10)	0.088 (9)	−0.001 (7)	0.008 (7)	−0.071 (8)
C22	0.113 (10)	0.080 (7)	0.116 (10)	0.008 (7)	−0.021 (8)	−0.075 (8)
C23	0.089 (7)	0.057 (5)	0.079 (7)	−0.002 (5)	−0.022 (6)	−0.033 (5)
C24	0.114 (10)	0.056 (6)	0.128 (11)	−0.026 (6)	−0.008 (8)	−0.039 (7)
C25	0.106 (9)	0.049 (5)	0.099 (9)	−0.029 (5)	0.006 (7)	−0.004 (6)
C26	0.072 (6)	0.041 (4)	0.050 (5)	−0.014 (4)	0.002 (4)	−0.006 (4)
C27	0.047 (4)	0.041 (4)	0.047 (5)	0.002 (3)	−0.015 (4)	−0.019 (3)
C28	0.038 (4)	0.054 (5)	0.036 (4)	0.004 (3)	−0.005 (3)	−0.017 (4)
Gd1	0.02026 (18)	0.0381 (2)	0.01841 (18)	−0.00571 (12)	0.00062 (11)	−0.00808 (13)
N1	0.068 (5)	0.094 (6)	0.053 (5)	0.011 (4)	−0.035 (4)	−0.034 (4)
N2	0.049 (4)	0.065 (4)	0.023 (3)	−0.016 (3)	−0.006 (3)	0.006 (3)
N3	0.041 (3)	0.051 (4)	0.036 (3)	0.003 (3)	0.011 (3)	−0.017 (3)
N4	0.046 (3)	0.042 (3)	0.030 (3)	−0.004 (3)	−0.002 (3)	−0.007 (3)
O1	0.052 (3)	0.048 (3)	0.026 (2)	−0.009 (2)	−0.013 (2)	−0.014 (2)
O2	0.043 (3)	0.059 (3)	0.046 (3)	−0.004 (2)	−0.002 (2)	−0.031 (3)
O3	0.082 (5)	0.163 (8)	0.098 (6)	0.055 (6)	−0.056 (5)	−0.072 (6)
O4	0.113 (6)	0.153 (8)	0.066 (5)	0.039 (6)	−0.060 (5)	−0.058 (5)
O5	0.060 (4)	0.104 (5)	0.033 (3)	−0.009 (3)	0.009 (3)	−0.035 (3)
O6	0.092 (6)	0.236 (11)	0.086 (6)	0.077 (7)	−0.041 (5)	−0.112 (7)
O7	0.027 (2)	0.044 (2)	0.024 (2)	−0.0110 (18)	−0.0016 (18)	−0.0002 (19)
O8	0.026 (2)	0.056 (3)	0.030 (3)	−0.014 (2)	−0.0062 (19)	0.006 (2)
O9	0.060 (4)	0.069 (4)	0.031 (3)	−0.023 (3)	0.008 (3)	0.012 (3)
O10	0.065 (5)	0.137 (7)	0.063 (5)	−0.040 (4)	−0.032 (4)	0.044 (4)
C16	0.032 (3)	0.045 (4)	0.014 (3)	−0.013 (3)	0.001 (2)	−0.009 (3)
O11	0.026 (2)	0.056 (3)	0.040 (3)	−0.012 (2)	0.008 (2)	−0.010 (2)
O12	0.040 (3)	0.054 (3)	0.032 (3)	−0.021 (2)	−0.005 (2)	0.004 (2)

### Geometric parameters (Å, °)

**Table:**

C1—O2	1.242 (8)	C19—H19	0.9300
C1—O1	1.256 (8)	C20—C28	1.405 (10)
C1—C2	1.500 (8)	C20—C21	1.411 (16)
C2—C3	1.390 (9)	C21—C22	1.322 (18)
C2—C7	1.391 (9)	C21—H21	0.9300
C3—C4	1.390 (10)	C22—C23	1.463 (16)
C3—H3	0.9300	C22—H22	0.9300
C4—C5	1.374 (10)	C23—C24	1.391 (16)
C4—N1	1.487 (10)	C23—C27	1.402 (12)
C5—C6	1.370 (10)	C24—C25	1.338 (16)
C5—H5	0.9300	C24—H24	0.9300
C6—C7	1.399 (9)	C25—C26	1.417 (13)
C6—C8	1.498 (10)	C25—H25	0.9300
C7—H7	0.9300	C26—N4	1.309 (10)
C8—O6	1.187 (10)	C26—H26	0.9300
C8—O5	1.308 (9)	C27—N4	1.380 (9)
C9—O8	1.243 (8)	C27—C28	1.445 (11)
C9—O7	1.268 (7)	C28—N3	1.348 (9)
C9—C10	1.519 (8)	Gd1—O12^i^	2.322 (5)
C10—C11	1.383 (9)	Gd1—O2^ii^	2.342 (4)
C10—C15	1.392 (8)	Gd1—O11^iii^	2.349 (4)
C11—C12	1.376 (9)	Gd1—O1	2.403 (4)
C11—H11	0.9300	Gd1—O8	2.442 (4)
C12—C13	1.396 (9)	Gd1—O7	2.499 (4)
C12—N2	1.479 (8)	Gd1—N3	2.571 (5)
C13—C14	1.383 (9)	Gd1—N4	2.611 (6)
C13—H13	0.9300	N1—O4	1.204 (9)
C14—C15	1.378 (8)	N1—O3	1.214 (10)
C14—C16	1.498 (8)	N2—O10	1.204 (9)
C15—H15	0.9300	N2—O9	1.220 (8)
C17—N3	1.324 (11)	O2—Gd1^ii^	2.342 (4)
C17—C18	1.401 (12)	O5—H5A	0.8200
C17—H17	0.9300	C16—O12	1.252 (8)
C18—C19	1.316 (16)	C16—O11	1.257 (7)
C18—H18	0.9300	O11—Gd1^iv^	2.349 (4)
C19—C20	1.434 (15)	O12—Gd1^i^	2.322 (5)
			
O2—C1—O1	125.4 (6)	C25—C24—C23	120.8 (10)
O2—C1—C2	116.8 (6)	C25—C24—H24	119.6
O1—C1—C2	117.8 (6)	C23—C24—H24	119.6
C3—C2—C7	120.1 (6)	C24—C25—C26	118.6 (10)
C3—C2—C1	118.6 (6)	C24—C25—H25	120.7
C7—C2—C1	121.3 (6)	C26—C25—H25	120.7
C4—C3—C2	118.1 (6)	N4—C26—C25	123.3 (9)
C4—C3—H3	121.0	N4—C26—H26	118.3
C2—C3—H3	121.0	C25—C26—H26	118.3
C5—C4—C3	122.9 (7)	N4—C27—C23	121.9 (9)
C5—C4—N1	118.7 (6)	N4—C27—C28	118.2 (6)
C3—C4—N1	118.4 (7)	C23—C27—C28	119.9 (8)
C6—C5—C4	118.5 (6)	N3—C28—C20	122.7 (8)
C6—C5—H5	120.8	N3—C28—C27	118.0 (6)
C4—C5—H5	120.8	C20—C28—C27	119.4 (8)
C5—C6—C7	120.7 (7)	O12^i^—Gd1—O2^ii^	76.27 (19)
C5—C6—C8	120.9 (6)	O12^i^—Gd1—O11^iii^	124.82 (17)
C7—C6—C8	118.3 (7)	O2^ii^—Gd1—O11^iii^	76.07 (18)
C2—C7—C6	119.7 (6)	O12^i^—Gd1—O1	75.73 (17)
C2—C7—H7	120.1	O2^ii^—Gd1—O1	126.15 (17)
C6—C7—H7	120.1	O11^iii^—Gd1—O1	83.73 (17)
O6—C8—O5	124.0 (7)	O12^i^—Gd1—O8	80.85 (15)
O6—C8—C6	124.2 (7)	O2^ii^—Gd1—O8	75.42 (17)
O5—C8—C6	111.8 (7)	O11^iii^—Gd1—O8	134.60 (16)
O8—C9—O7	122.3 (5)	O1—Gd1—O8	141.63 (17)
O8—C9—C10	118.4 (5)	O12^i^—Gd1—O7	132.49 (15)
O7—C9—C10	119.1 (5)	O2^ii^—Gd1—O7	81.73 (16)
C11—C10—C15	120.8 (5)	O11^iii^—Gd1—O7	88.64 (15)
C11—C10—C9	121.4 (5)	O1—Gd1—O7	147.63 (16)
C15—C10—C9	117.6 (5)	O8—Gd1—O7	52.84 (14)
C12—C11—C10	117.3 (6)	O12^i^—Gd1—N3	84.99 (19)
C12—C11—H11	121.4	O2^ii^—Gd1—N3	145.6 (2)
C10—C11—H11	121.4	O11^iii^—Gd1—N3	137.47 (19)
C11—C12—C13	123.4 (6)	O1—Gd1—N3	74.72 (18)
C11—C12—N2	119.3 (6)	O8—Gd1—N3	73.32 (19)
C13—C12—N2	117.3 (6)	O7—Gd1—N3	90.53 (17)
C14—C13—C12	117.9 (6)	O12^i^—Gd1—N4	138.41 (18)
C14—C13—H13	121.1	O2^ii^—Gd1—N4	144.26 (19)
C12—C13—H13	121.1	O11^iii^—Gd1—N4	74.83 (18)
C15—C14—C13	120.1 (5)	O1—Gd1—N4	70.35 (17)
C15—C14—C16	119.7 (5)	O8—Gd1—N4	112.27 (17)
C13—C14—C16	120.2 (5)	O7—Gd1—N4	77.29 (16)
C14—C15—C10	120.5 (6)	N3—Gd1—N4	63.6 (2)
C14—C15—H15	119.8	O4—N1—O3	124.4 (8)
C10—C15—H15	119.8	O4—N1—C4	117.7 (8)
N3—C17—C18	123.5 (10)	O3—N1—C4	117.9 (7)
N3—C17—H17	118.2	O10—N2—O9	122.6 (6)
C18—C17—H17	118.2	O10—N2—C12	119.1 (6)
C19—C18—C17	119.6 (10)	O9—N2—C12	118.3 (6)
C19—C18—H18	120.2	C17—N3—C28	117.6 (6)
C17—C18—H18	120.2	C17—N3—Gd1	121.2 (5)
C18—C19—C20	119.8 (8)	C28—N3—Gd1	119.7 (5)
C18—C19—H19	120.1	C26—N4—C27	117.6 (7)
C20—C19—H19	120.1	C26—N4—Gd1	124.1 (5)
C28—C20—C21	119.8 (10)	C27—N4—Gd1	116.9 (5)
C28—C20—C19	116.8 (9)	C1—O1—Gd1	130.5 (4)
C21—C20—C19	123.4 (9)	C1—O2—Gd1^ii^	153.7 (4)
C22—C21—C20	121.3 (10)	C8—O5—H5A	109.5
C22—C21—H21	119.4	C9—O7—Gd1	90.7 (3)
C20—C21—H21	119.4	C9—O8—Gd1	94.0 (4)
C21—C22—C23	122.2 (10)	O12—C16—O11	124.9 (6)
C21—C22—H22	118.9	O12—C16—C14	117.3 (5)
C23—C22—H22	118.9	O11—C16—C14	117.8 (6)
C24—C23—C27	117.7 (10)	C16—O11—Gd1^iv^	127.2 (4)
C24—C23—C22	124.9 (10)	C16—O12—Gd1^i^	161.3 (4)
C27—C23—C22	117.4 (11)		

Symmetry codes: (i) −*x*+1, −*y*, −*z*+1; (ii) −*x*+2, −*y*, −*z*+1; (iii) *x*+1, *y*, *z*; (iv) *x*−1, *y*, *z*.

### Hydrogen-bond geometry (Å, °)

**Table:**

*D*—H···*A*	*D*—H	H···*A*	*D*···*A*	*D*—H···*A*
O5—H5A···O7^v^	0.82	2.03	2.737 (7)	145

Symmetry codes: (v) *x*, *y*, *z*+1.
